# IL-17A Induces Pendrin Expression and Chloride-Bicarbonate Exchange in Human Bronchial Epithelial Cells

**DOI:** 10.1371/journal.pone.0103263

**Published:** 2014-08-20

**Authors:** Kelly M. Adams, Valsamma Abraham, Daniel Spielman, Jay K. Kolls, Ronald C. Rubenstein, Gregory E. Conner, Noam A. Cohen, James L. Kreindler

**Affiliations:** 1 Department of Pediatrics, Children's Hospital of Philadelphia, Philadelphia, Pennsylvania, United States of America; 2 Department of Pediatrics, Perelman School of Medicine at the University of Pennsylvania, Philadelphia, Pennsylvania, United States of America; 3 School of Arts and Sciences, University of Pennsylvania, Philadelphia, Pennsylvania, United States of America; 4 Departments of Pediatrics and Immunology, University of Pittsburgh School of Medicine, Pittsburgh, Pennsylvania, United States of America; 5 Department of Cell Biology, University of Miami Miller School of Medicine, Miami, Florida, United States of America; 6 Department of Otorhinolaryngology, Perelman School of Medicine at the University of Pennsylvania, Philadelphia, Pennsylvania, United States of America; University of Tübingen, Germany

## Abstract

The epithelium plays an active role in the response to inhaled pathogens in part by responding to signals from the immune system. Epithelial responses may include changes in chemokine expression, increased mucin production and antimicrobial peptide secretion, and changes in ion transport. We previously demonstrated that interleukin-17A (IL-17A), which is critical for lung host defense against extracellular bacteria, significantly raised airway surface pH in vitro, a finding that is common to a number of inflammatory diseases. Using microarray analysis of normal human bronchial epithelial (HBE) cells treated with IL-17A, we identified the electroneutral chloride-bicarbonate exchanger Pendrin (SLC26A4) as a potential mediator of this effect. These data were verified by real-time, quantitative PCR that demonstrated a time-dependent increase in Pendrin mRNA expression in HBE cells treated with IL-17A up to 48 h. Using immunoblotting and immunofluorescence, we confirmed that Pendrin protein expression is increased in IL-17 treated HBE cells and that it is primarily localized to the mucosal surface of the cells. Functional studies using live-cell fluorescence to measure intracellular pH demonstrated that IL-17A induced chloride-bicarbonate exchange in HBE cells that was not present in the absence of IL-17A. Furthermore, HBE cells treated with short interfering RNA against Pendrin showed substantially reduced chloride-bicarbonate exchange. These data suggest that Pendrin is part of IL-17A-dependent epithelial changes and that Pendrin may therefore be a therapeutic target in IL-17A-dependent lung disease.

## Introduction

IL-17A plays a central role in multiple facets of the immune response of the lung. Its activity is critical for host defense against extracellular bacteria including Haemophilus influenzae [1], [2] and Staphylococcus aureus [3], [4], [5]. For example, in patients with Hyper-IgE syndrome, who lack ThIL-17 cells, *Sa* skin and lung infections are common [5]. In mice, loss of ThIL-17-mediated immunity after influenza infection predisposes to *Sa* pneumonia [6]. Notably, ThIL-17 cells, the main producers of IL-17A, are found in airways submucosa early in the course of cystic fibrosis (CF) [7], and IL-17A levels are increased in sputum during pulmonary exacerbations of CF and return to normal only after treatment [1], [8]. In addition to a role in host defense, IL-17A and its related family members have a role in the pathophysiology of asthma. In particular, IL-17A is thought to contribute to the neutrophilic airways inflammation seen in severe asthmatics [9]. Therefore, IL-17A-mediated immune functions are potential targets for therapeutic manipulation in a number of respiratory diseases.

Because of the importance of IL-17A in lung host defense, several studies have investigated its effects on airway epithelial cells. The IL-17 receptor is highly expressed on human bronchial epithelial (HBE) cells [8], [10], in which IL-17A induces transcription of airway mucins [11], antimicrobial peptides [12], and pro-inflammatory cytokines and chemokines that favor production and influx of neutrophils into the lung [13], [14]. Epithelial ion transport is closely linked to mucin biology [15], [16], antimicrobial peptide function [17], and inflammation [18], [19]. Therefore, we hypothesized that IL-17A may alter epithelial ion transport properties. We previously found that IL-17A induced CFTR-dependent HCO_3_^−^ secretion in HBE cells [20]. We also made the observation that IL-17A increased apical surface pH (pH_ASL_) in the absence of exogenous stimuli and without affecting baseline short-circuit current, suggesting that it promoted an electroneutral mechanism that changed surface pH [20].

The simplest explanation for the observed increase in pH_ASL_ in IL-17A-stimulated HBE cells would be either decreased H^+^ secretion or increased HCO_3_^−^ secretion across the apical membrane of the cells. Microarray analysis had shown that IL-17A strongly upregulated Pendrin expression in HBE cells (data from this array were previously published [1]). Pendrin (SLC26A4) is an electroneutral, HCO_3_^−^-secreting protein, and, thus, a good candidate to mediate the observed effect on pH_ASL_. Pendrin is a member of the SLC26A family of Cl^−^-dependent anion transporters that are found at the apical plasma membrane of many epithelia. The SLC26A family of integral membrane proteins share common signal transduction and anti-sigma factor (STAS) domains that can interact with and regulate CFTR [21]. SLC26A6 (PAT1) mediates CFTR-regulated HCO_3_^−^ secretion in the pancreas [22]. SLC26A9 is expressed in human airways, but appears to primarily act as a Cl^−^ channel rather than a Cl^−^/HCO_3_^−^ exchanger [23]. While SLC26A3, which we do identify in HBE cells at the mRNA level, is reported to mediate CFTR-dependent Cl^−^/HCO_3_^−^ exchange in immortalized tracheal cells [24], others did not detect SLC26A3 mRNA outside of the gastrointestinal tract in ribonuclease protection assays [25]. Furthermore, our data suggest that IL-17A reduces SLC26A3 expression, which would be expected to cause a decrease in net HCO_3_^−^ transport. Therefore, SLC26A3 is not a good candidate to mediate the effects that we observed.

Pendrin is an electroneutral, Cl^−^/anion exchanger that can transport HCO_3_^−^ [26], [27], iodide (I^−^) [28], [29], and thiocyanate (SCN^−^) [30]. Within the Cl^−^/HCO_3_^−^ exchangers of the SLC26A family Pendrin is unique in its lack of a c-terminal PDZ domain [31], the absence of which may explain its CFTR independence. Pendrin is expressed in many epithelia, including those from lung, thyroid, inner ear, and kidney. Genetic mutations in Pendrin cause Pendred syndrome (OMIM #274600), a disease characterized by congenital deafness, goiter, and thyroid hormone abnormalities [28]. The role of Pendrin in human lung physiology or pathophysiology is not yet known. To date, lung disease has not been described in Pendred syndrome patients.

Therefore, we tested the hypothesis that IL-17A specifically increased the expression and function of Pendrin in HBE cells.

## Materials and Methods

### Cell culture

Normal HBE cells purchased from Lonza (Walkersville, MD) were cultured to maturity at air-liquid interface as previously described (14) (17, 28, 59–60).

Epithelial cells were isolated from airways of CF patients at the time of transplant following protocols (Function and Differentiation of the Airway Epithelium) approved by the University of Miami Institutional Review Board. Written informed consent was obtained prior to the collection of the samples.

### Real-time PCR

RNA was isolated using Qiagen's RNeasy Kit. cDNA was generated with Applied Biosystems High Capacity Reverse Transcription cDNA Kit. Real-time PCR was performed using pre-designed primer-probe combinations with Taqman reagents (both Applied Biosystems). Data were analyzed by comparative threshold cycle (C_T_) analysis using GAPDH as the control and normalizing all values to the mean of the control group [32].

#### RNA-seq sample preparation

Total RNA from HBE cells (1–4 ug) that had or had not been stimulated with cytokines was used as starting material for deep sequencing using the Illumina TruSeq RNA Sample Preparation v2 Guide. Briefly, mRNA was purified with oligo-dT beads, fragmented with magnesium and heat-cataylzed hyrdolysis, and used as a template for first- and second-strand cDNA synthesis with random primers. The cDNA 3′ ends were adenylated, followed by adaptor ligation and a 15-cycle PCR to enrich DNA fragments. Quantification of cDNA libraries was performed by using Kapa Biosystems primer premix kit with Illumina-compatible DNA primers. For cluster generation, the TruSeq SR Cluster Kit v2-cBot-GA was used, and cDNA libraries were loaded onto the flow cell at a final concentration of 6pM. Single-read sequencing was performed on the Illumina Genome Analyzer II.

### Immunoblotting

Vehicle or IL-17A stimulated HBE cells were lysed with RIPA buffer plus 10 mM Na Orthovandate, 1 mM PMSF, and protease inhibitors (Roche). Lysates were sonicated on ice and centrifuged for clarification. 30 µg total protein were separated by PAGE then transferred to PVDF membranes. Immunoblotting was performed with the anti-Pendrin antibody H-195 (1∶500, Santa Cruz) followed by a HRP-linked goat anti-rabbit antibody (Santa Cruz). After detection of Pendrin, membranes were stripped and actin detected as a loading control.

### Immunofluorescence

HBE cells were fixed with 4% paraformaldehyde and permeabilized with 0.3% Triton-X100 prior to blocking (1% BSA/5% normal goat). Primary antibody was applied overnight at 4°C (1∶500 Pendrin; 1∶1000 Type IV Tubulin: Abcam). Secondary antibody was applied for 1 h at RT (1∶500 goat anti-rabbit IgG Alexa fluor 594 (Pendrin) plus 1∶750 goat anti-mouse IgG Alexa fluor 488 (Type IV Tubulin). Nuclei were counterstained with Hoechst at 1∶1000.

### Fluorescence microscopy

HBE cells loaded with the pH-sensitive dye SNARF-5/6 (Invitrogen, Carlsbad, CA) [20] were mounted in a specially designed holder (Bioptechs, Butler, PA) on the stage of an inverted fluorescence microscope. Cells were bathed on the serosal side and perfused on the mucosal side. Mucosal solution changes were made using a pinch-valve system (Warner Instruments, Hamden, CT). Intracellular dye was excited with light from a xenon lamp filtered with a 480/20 nm band-pass filter. The ratio of fluorescence emissions at 640 nm and 580 nm was captured every 10 or 20 s using a Hamamatsu ORCA-ER camera and SlideBook software (Intelligent Imaging Innovations, Inc.). Data were stored on a computer hard drive and analyzed using Prism 5 (GraphPad Software, San Diego, CA). Conversion from F640/F580 was performed after calibration of intracellular pH (pHi) at 6.8, 7.2, and 7.6.

siRNA inhibition of Pendrin expression: siRNA duplexes were introduced into epithelial cells using a modification of previously published methods [30].

### Chemicals

Recombinant human IL-17A (R&D Systems) was dissolved in 4 mM HCl and used at 50 ng/ml. DIDS (Invitrogen) and niflumic acid (Sigma) were dissolved in DMSO as 1000× stocks and added to the mucosal, chloride-free solution at 100 µM each. NF-κB inhibitor II (JSH-23) was dissolved as a 1000× stock in DMSO and used at 20 µM.

### Statistical analysis

Values are mean ± S.E.M. Statistical were analyzed with Prism 5, using Student's *t*-test, two-way ANOVA with Bonferroni posttests, or repeated measures ANOVA as appropriate. Significance was defined as a p≤0.05.

Data from Gene Expression Omnibus microarray accession code, GSE10240, are referenced in the results section.

## Results

### IL-17A directly and specifically induces Pendrin mRNA expression

**Figure 1:**
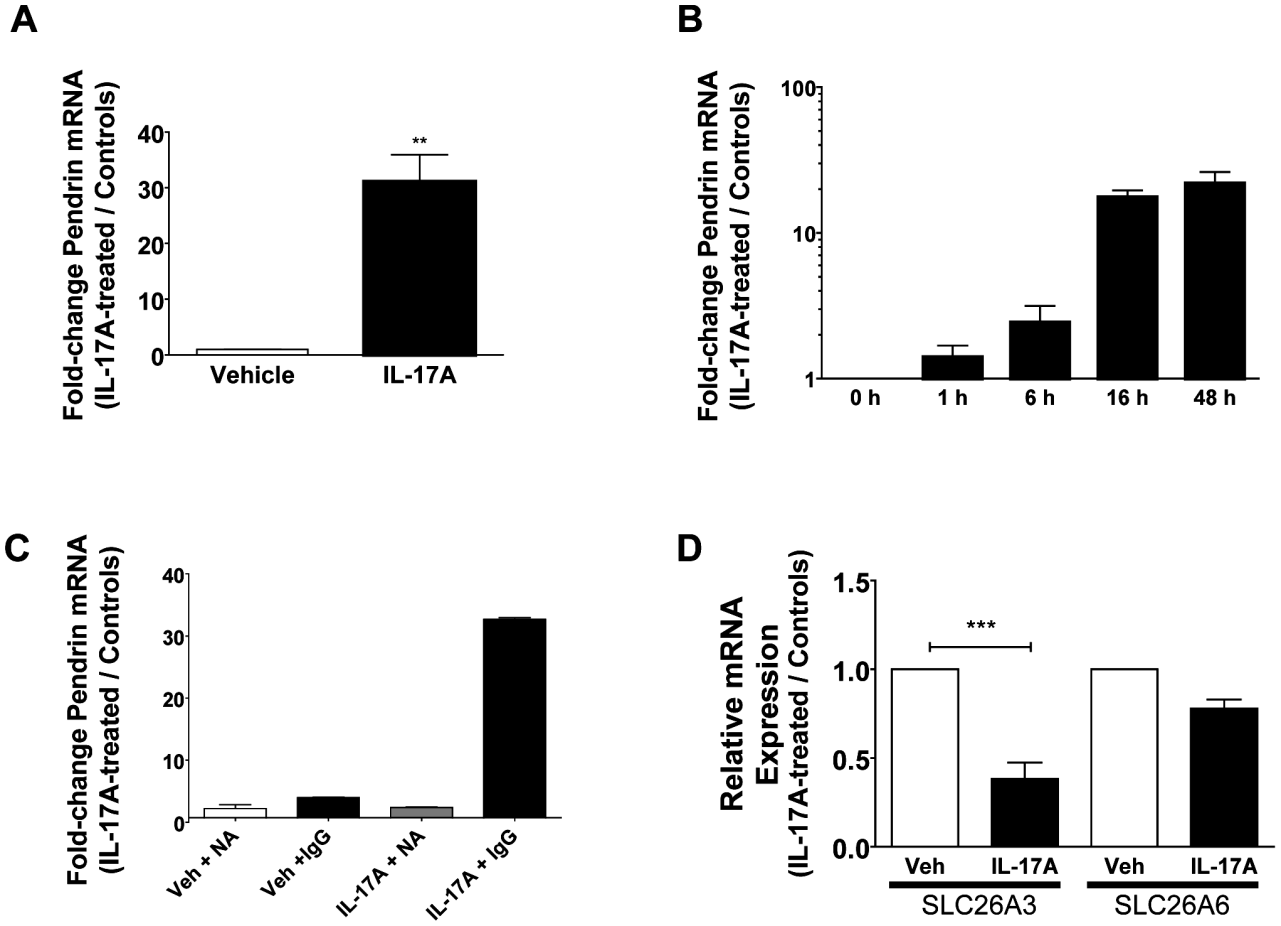
IL-17A-dependent induction of Pendrin mRNA. **A.** Mature, well-differentiated HBE cells were stimulated with IL-17A (50 ng/ml, 48 h) prior to collection of total RNA, reverse-transcription, and analysis of Pendrin mRNA expression by quantitative PCR (n = 5 inserts, ** p<0.01). **B.** IL-17A increases Pendrin mRNA in a time-dependent fashion (Note logarithmic scale, n = 2 inserts per time point, one from each of 2 donors compared to its own control). **C.** HBE cells were stimulated with IL-17A (50 ng/ml, 48 h) or vehicle (PBS) after which conditioned media were collected and used to stimulate naïve cells. Conditioned media were treated with either an IL-17A neutralizing antibody (NA) or an isotype control IgG (IgG) (n = 2 inserts per condition, one from each of two donors compared to its own vehicle control). D. Quantitative PCR for SLC26A3 (DRA) and SLC26A6 (PAT1) in the presence and absence of IL-17A (n = 3 inserts per condition).

Our microarray data strongly suggested that IL-17A increased Pendrin expression in normal HBE cells. To verify this by quantitative PCR, we stimulated HBE cells with IL-17A at 50 ng/ml for 48 h, which were the conditions used in the microarray and in our previously published data [20]. Following stimulation, real-time PCR was performed to quantify the relative change in Pendrin expression in IL-17A-treated cells compared with vehicle controls using GAPDH as the reference gene. These data confirmed that IL-17A significantly increased Pendrin mRNA expression (Figure 1a) in a time-dependent manner, with increases appearing by 6 h and maximal at 48 h, the longest time point measured (Figure 1b).

Previous data demonstrated that IL-17A can signal through autocrine/paracrine mechanisms to regulate mucin transcription [33]. To test if this mechanism was necessary for IL-17A-mediated Pendrin expression we performed an experiment in which naïve HBE cells were treated with conditioned medium from cells treated with IL-17A for 48 h. Conditioned media were treated either with an IL-17A neutralizing antibody (317-ILB, R&D Systems) or with an isotype control antibody. Naïve cells fed with conditioned medium treated with control (non-neutralizing) antibody demonstrated significant increases in Pendrin expression after 24 h. Conversely, cells treated with IL-17A neutralized conditioned medium did not show significant increases in Pendrin expression (Figure 1c). Multiplex cytokine analysis of conditioned media confirmed that IL-17A induced G-CSF production [8], but failed to show increased IL-6 production in IL-17A-treated HBE cells (data not shown). Together, these data support the conclusion that IL-17A alone is sufficient for increased Pendrin expression under our experimental conditions. Although IL-17A is sufficient to promote Pendrin expression in HBE cells, other cytokines including interferon-γ and IL-13 also appear to increase Pendrin RNA expression (Figure S1).

Pendrin is a member of the SLC26A family of anion exchangers [34]. Therefore, we wanted to investigate whether IL-17A increased the expression of other SLC26A Cl^−^/HCO_3_^−^ exchangers that are expressed in HBE cells. In cells treated with IL-17A for 48 h there was a significant decrease in SLC26A3 (DRA) expression and no significant change in SLC26A6 expression (Figure 1d), suggesting that the effect of IL-17A on Pendrin expression is not a non-specific effect seen across SLC26A family members. Additionally, because some members of the SLC26 family interact with the cystic fibrosis transmembrane conductance regulator (CFTR), we measured changes in CFTR mRNA after 48 h of IL-17A stimulation (50 ng/ml). CFTR expression increased by an average of 1.8-fold (range 0.97–2.5) in IL-17A-treated cells relative to vehicle controls (n = 7 inserts, representing 2 donors; data not shown).

### IL-17A induces Pendrin protein expression at the apical membrane of normal HBE cells

**Figure 2:**
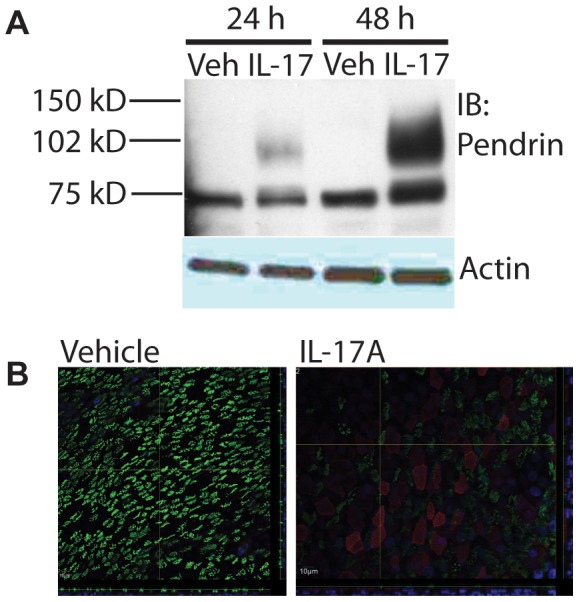
IL-17A induces Pendrin protein expression at the apical plasma membrane of normal HBE cells. **A.** Immunoblot of Pendrin from total cell lysates at 24 h and 48 h of IL-17A stimulation. **B.** Confocal immunofluorescence images of vehicle and IL-17A stimulated HBE cells. Note that Pendrin (red) is only detected in IL-17A stimulated cells and is primarily localized to non-ciliated cells, which lack of Type IV Tubulin (green). Images are z-stacks with 0.5 micron sections from the Transwell support to the tips of the cilia. For each set of images, the main image is a single slice from the z-stack. Below is an x–z projection and to the right is a y–z projection. The yellow lines indicate the location from which the projections are taken.

To demonstrate that the observed increases in Pendrin mRNA correlated with increased protein expression, we performed immunoblotting using a commercially available anti-Pendrin antibody. Consistent with its effect on Pendrin transcription, IL-17A significantly increased Pendrin protein expression in a time-dependent fashion with Pendrin protein expression appearing to be greater at 48 h compared to 24 h (Figure 2a). Because the antibody we used identified a dominant band at the expected size for Pendrin [27], we performed immunofluorescence on mature, differentiated HBE cells treated with either vehicle or IL-17A. Confocal imaging confirmed greater Pendrin expression in IL-17A-treated cells and revealed that Pendrin is expressed at the mucosal surface of the cells (Figure 2b, right panel). This is best appreciated in the main XY image where Pendrin staining is present in the same 0.5 micron section as discreet cilia that are stained with Type IV Tubulin. This is also demonstrated in the XZ sections, though it is more difficult to appreciate because these cell preparations were only about 10 microns thick. Pendrin appeared to be expressed primarily in non-ciliated cells, i.e. those cells in Figure 2b that do not have Type IV Tubulin staining, which identifies cilia. Notably, vehicle controls did not demonstrate any immunofluorescence signal even in the presence of primary antibody (Figure 2b, left panel), nor did IL-17A-treated cells exposed only to secondary antibody (Figure S2, panel g). Similar results were obtained with two different, commercially available Pendrin antibodies (Figure S2, panels a–c and d–f). For these antibodies, synthetic antigenic peptide was available, and co-incubation of the primary antibody with the peptide reduced immunofluorescence signal (Figure S2, panels c and f).

### IL-17A induces CFTR-independent Cl^−^/HCO_3_^−^ exchange at the mucosal surface of HBE cells

**Figure 3:**
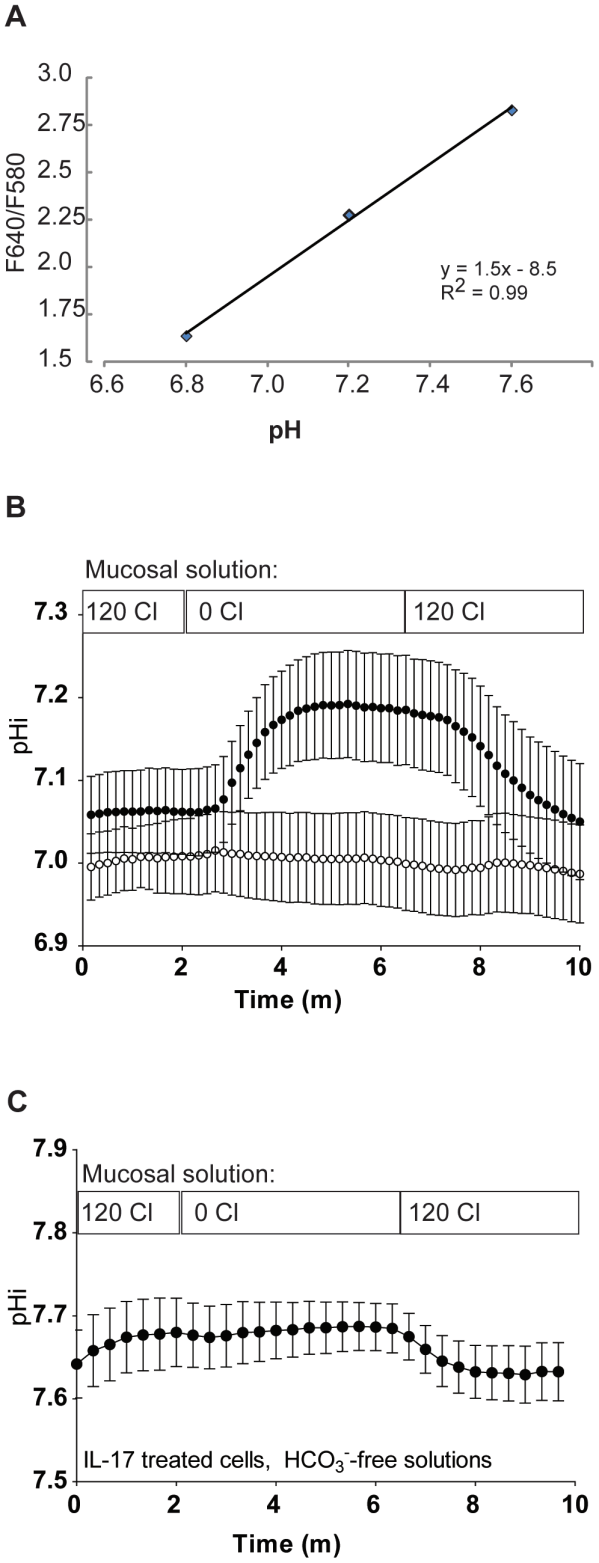
IL-17A induces Cl^−^/HCO_3_^−^ exchange at the apical membrane of normal HBE cells. **A.** Calibration curve of pHi versus F640/580. An insert of HBE cells was loaded with SNARF-5/6-AM and then incubated in high-potassium buffer (pH 6.8, 7.2, or 7.6) containing 20 µM each valinomycin and nigericin for 40 minutes. Five serial measurements over 2 minutes were taken for each filter and the average used to calculate a single calibration point for each pH. Linear fit was performed in Microsoft Excel. **B**. Intracellular pH changes in response to chloride removal from the apical perfusate (open circles: vehicle, n = 6 inserts; shaded circles: IL-17A (50 ng/ml for 48 h, n = 5 inserts). **C**. Intracellular pH changes in response to chloride removal from the apical perfusate in the absence of soluble HCO_3_^−^ and CO_2_ (only IL-17A treated cells are shown). Please see the results section in the text for a complete discussion of two-way ANOVA applied to 3b.

The finding of increased Pendrin expression at the apical membrane of HBE cells prompted us to look for functional evidence of its expression. To accomplish this goal, we loaded HBE cells with the pH-sensitive dye SNARF-5/6 and mounted them in a specially-designed perfusion apparatus that allowed for differential perfusion of the mucosal and serosal surfaces of the cells. This apparatus was placed on the stage of an inverted fluorescence microscope for live-cell imaging. To probe the HCO_3_^−^ exit pathway across the apical membrane, we established a Cl^−^ gradient that promoted movement of Cl^−^ out of the cell across the apical membrane. To accomplish this, the apical membrane was initially perfused with solution containing both Cl^−^ and HCO_3_^−^ (described in Methods S1). After a 2–3 minute period of stabilization during which fluorescence readings were made, the mucosal solution was changed to one in which Cl^−^ salts were replaced with equimolar gluconate salts. In IL-17A-treated cells this resulted in a rapid increase in pHi (Figure 3b) that was not seen in controls. By two-way ANOVA with Bonferroni post-test correction, there were no statistically significant differences at individual time points when comparing IL-17A-treated cells and controls. However, when the change in pHi from baseline was examined by subtracting the average pHi from time 0–2 minutes from that of the average pHi from time 5–6 minutes, there was a statistically greater change in the IL-17A-treated cells (0.13±0.02 pH units) than in controls (0.001±0.02 pH units).

This change was dependent on the presence of HCO_3_^−^ in the bath solutions (Figure 3c), supporting the notion that the pH change was due to Cl^−^/HCO_3_^−^ exchange as opposed to changes in H^+^ transport, though we note in these experiments that the starting intracellular pH was significantly higher than in the presence of CO_2_. In fact, the baseline pH was close to the upper limit of linearity in our assay (Figure 3a), which may have limited our ability to detect a rise in pHi in the absence of HCO_3_^−^. There was a small decrease in pHi when Cl^−^ was replaced in the apical bath solution, consistent with loss of intracellular base equivalents.

**Figure 4:**
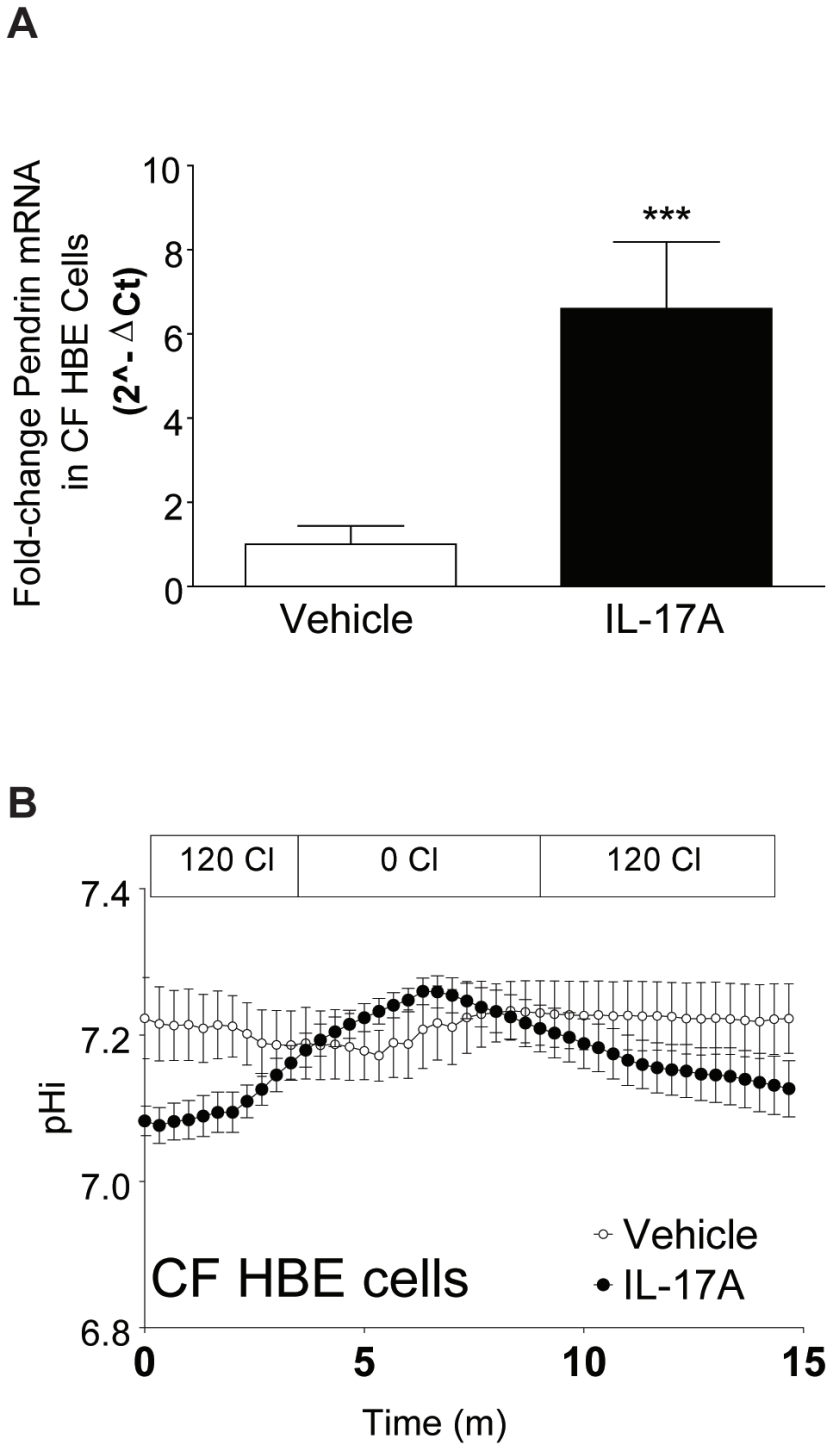
IL-17A increases Pendrin and Cl^−^/HCO_3_^−^ exchange in CF HBE cells. **A.** Pendrin mRNA is increased in CF HBE cells treated with IL-17A (shaded bar) (n = 9 inserts from 2 donors, *** p<0.01 compared to vehicle controls (open bar)). **B.** Consistent with the increase in Pendrin expression, apical membrane Cl^−^/HCO_3_^−^ is also increased in CF HBE cells treated with IL-17A (closed circles, n = 3 inserts).

Pendrin, which we hypothesize is responsible for IL-17A-induced Cl^−^/HCO_3_^−^ exchange, is a CFTR-independent exchanger [30], [35], as opposed to SLC26A3 and SLC26A6, which are CFTR-dependent [21]. To explore whether IL-17A-induced Cl^−^/HCO_3_^−^ exchange was CFTR-dependent or independent, we stimulated CF HBE cells with IL-17A (50 ng/ml, 48 h) and evaluated for both Pendrin expression and Cl^−^/HCO_3_^−^ exchange. Similar to results in normal HBE cells, IL-17A induced Pendrin expression (Figure 4a) and induced Cl^−^/HCO_3_^−^ exchange (Figure 4b) in CF HBE cells.

### siRNA inhibition of Pendrin expression reduces IL-17A-induced Cl^−^/HCO_3_^−^ exchange

**Figure 5:**
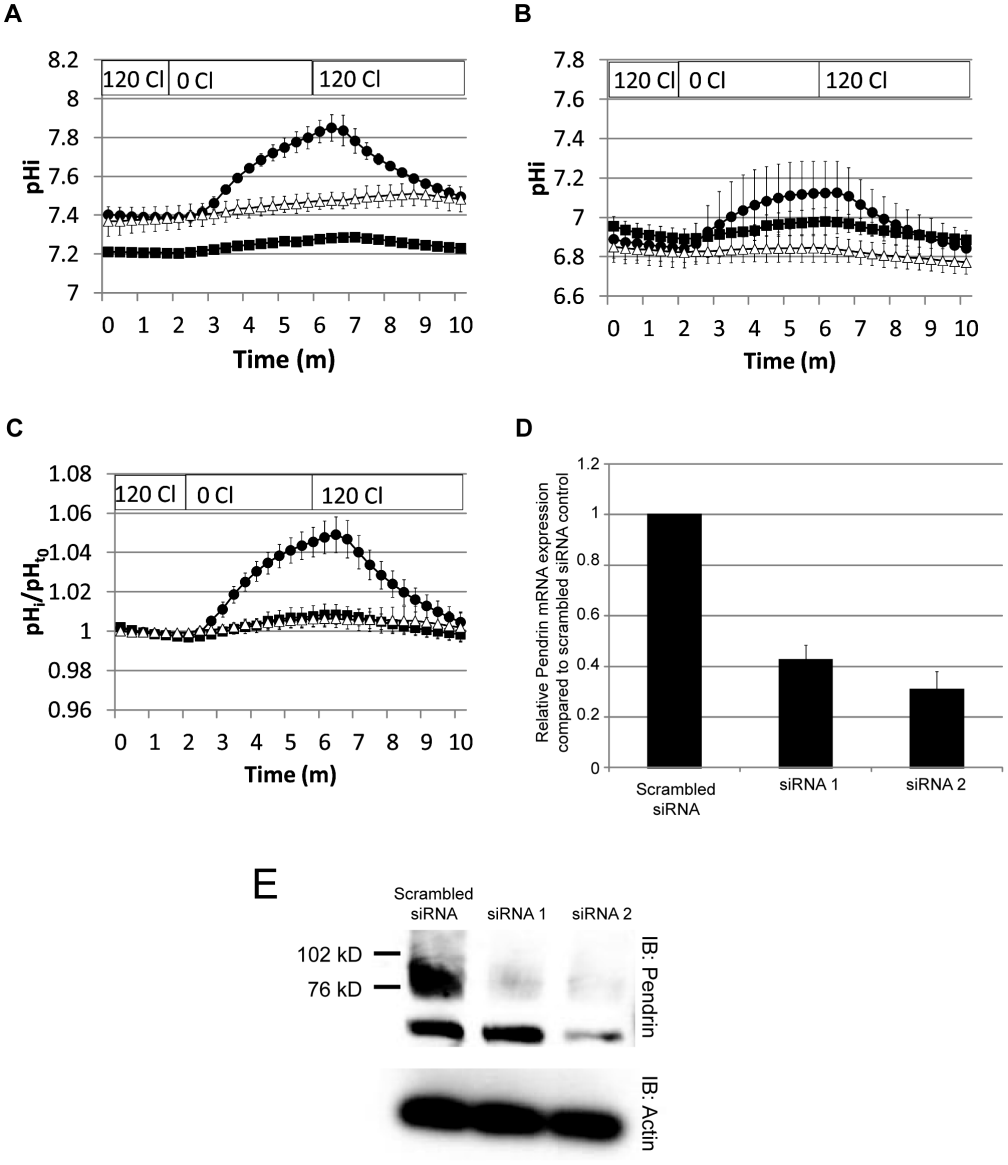
Inhibition of Pendrin expression by siRNA reduces IL-17A-induced Cl^−^/HCO_3_^−^ exchange. **A. and B.** Normal HBE cells from two different donors were transfected with either non-targeting (scrambled) siRNA (closed circles) or one of two Pendrin-targeting siRNA from Invitrogen: HSS 107794 (siRNA 1, closed squares) and HSS 107796 (siRNA 2, open triangles). After 14 days, cells were tested for Cl^−^/HCO_3_^−^ exchange. Panels A and B represent data from the individual donor. **C.** To account for differences in baseline pHi, pH was normalized to the mean of the first minute (pH_t0_) and data were then combined. Normalized mean data encompassing both donors are shown in panel C. **D.** Quantitative PCR measuring Pendrin mRNA expression in targeting siRNA-treated cells compared with scrambled control siRNA-treated cells. E. Immunoblot comparing Pendrin protein expression in targeting siRNA-treated cells compared with scrambled control siRNA-treated cells.

In order to test more directly the hypothesis that Pendrin expression is required for IL-17A-induced Cl^−^/HCO_3_^−^ exchange, normal HBE cells were transfected with one of two siRNA molecules directed against Pendrin or with a non-targeting control siRNA. Each of the two siRNA sequences was tested in two tissue donors. Individually, siRNA treatment resulted in decreased Cl^−^/HCO_3_^−^ exchange in IL-17A-treated cells (Figure 5a and 5b) when compared to controls. Differences in baseline pHi between the two experiments made combining the raw data difficult. Therefore, pHi was normalized to the average of the pHi readings over the first minute. Data from the two experiments were then combined, and show a consistent and robust diminution of Cl^−^/HCO_3_^−^ exchange in siRNA-treated cells when compared with controls (Figure 5c). Real-time PCR (Figure 5d) and immunoblotting (Figure 5e) confirmed that siRNA treatment decreased Pendrin expression compared to controls.

### IL-17A induces Pendrin expression via NF-κB

**Figure 6:**
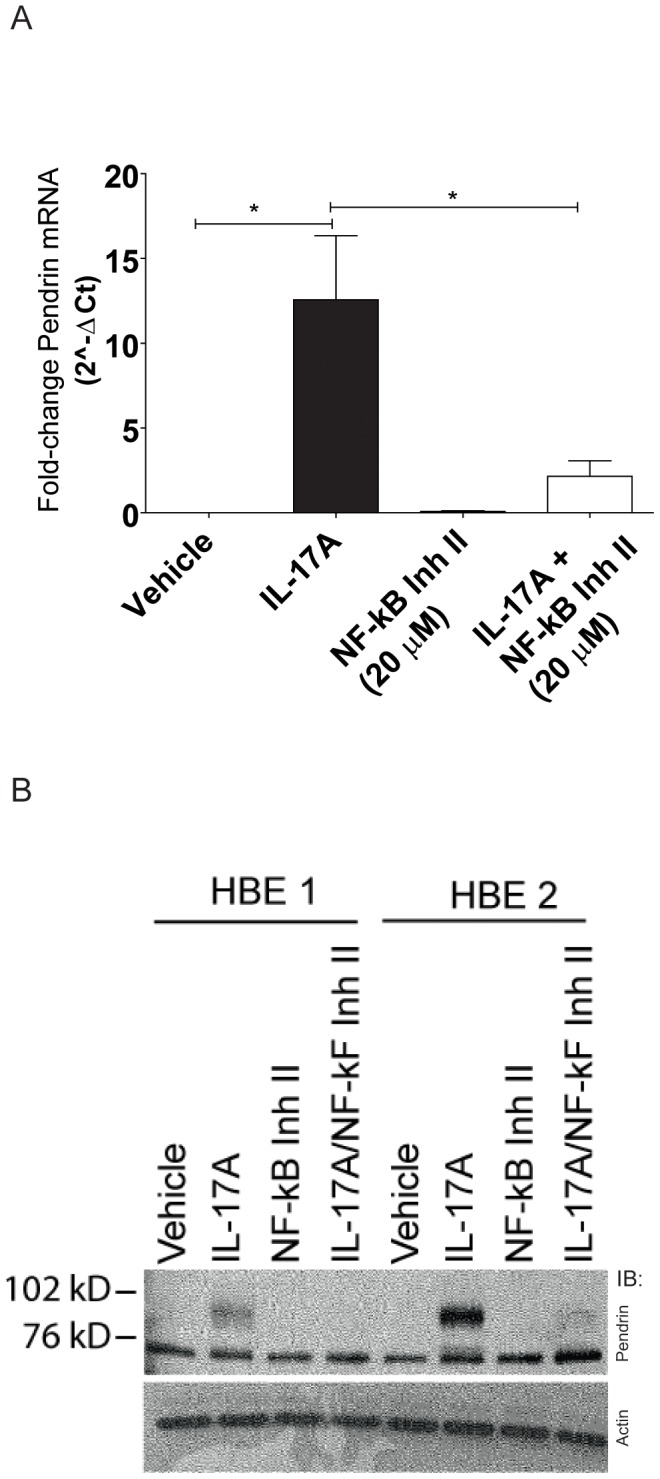
IL-17A-induced Pendrin expression is dependent on NF-κB. Cells were incubated with NF-κB inhibitor II (20 µM) for 6 hours and then with NF-κB inhibitor II and IL-17A (10 ng/ml) for 24 h prior to analysis. Quantitative PCR (A) and immunoblotting (B) from normal HBE cells demonstrating that inhibition of NF-κB prevents IL-17A induced Pendrin expression.

IL-17A signals to HBE cells through a number of pathways, including canonical NF-κB signaling and through JAK signaling [36]. Because the IL-17A-induced epithelial changes in antimicrobial peptide production and mucin transcription are driven by NF-κB [12], [37], we hypothesized that airway epithelial Pendrin expression would also be dependent on NF-κB signaling. Therefore, we pre-treated HBE cells with vehicle (DMSO) or NF-κB inhibitor II (20 µM, JSH-23, Calbiochem) for 1 h prior to stimulation with IL-17A. DMSO-treated cells stimulated with IL-17A showed robust increases in Pendrin mRNA (Figure 6A) and protein (Figure 6B) expression, whereas NF-κB inhibitor II-treated cells stimulated with IL-17A were not different from unstimulated controls.

## Discussion

IL-17A has numerous effects on the biology and physiology of airway epithelial cells, including effects on ion transport. Previously, we demonstrated that IL-17A stimulated CFTR-dependent HCO_3_^−^ secretion [20]. As part of those studies, we measured resting airway surface pH (pH_ASL_) and observed that IL-17A increased pH_ASL_ in an electroneutral, CFTR-independent fashion. The current studies were undertaken to explore the mechanism by which IL-17A might have this effect. We hypothesized that a rise in pH_ASL_ would reflect a change in net ion transport rather than changes in other buffering systems. This hypothesis was consistent with microarray data comparing vehicle-treated HBE cells with cells that were stimulated with IL-17A, IL-22, or a combination of the two that suggested IL-17A-stimulated HBE cells expressed more Pendrin, a HCO_3_^−^ secreting Cl^−^/HCO_3_^−^ exchanger, mRNA than did vehicle controls [1].

We first confirmed our microarray observations using quantitative PCR, demonstrating that there is a time-dependent increase in Pendrin mRNA expression in IL-17A stimulated HBE cells without increases in mRNA expression of other SLC26A Cl^−^/HCO_3_^−^ exchangers expressed in HBE cells. These findings are in agreement with others who have found that Th2 cytokines induce Pendrin expression, but not other SLC26A family members [30]. Associated with the increase in Pendrin mRNA that we observed, IL-17A stimulated HBE cells demonstrate a time-dependent increase in Pendrin protein expression. Moreover, Pendrin protein expression can be localized by immunofluorescence to the apical domain of the cells. Although the Pendrin antibody used was not monospecific and, therefore, immunofluorescence results may represent nonspecific binding, we do not favor this interpretation because the dominant band detected in immunoblots was of the anticipated molecular weight and because multiple Pendrin antibodies produced similar results (Figure S2). Therefore, we concluded that IL-17A increased Pendrin expression at the apical membrane in normal HBE cells, and we undertook experiments to test the hypothesis that IL-17A would also increase Cl^−^/HCO_3_^−^ exchange in these cells.

The experimental system we chose to investigate apical Cl^−^/HCO_3_^−^ exchange was one in which Cl^−^ is rapidly replaced in the mucosal solution by gluconate, an impermeant anion. Cl^−^ removal from the mucosal bath creates a large concentration gradient for Cl^−^ from serosal (120 mM Cl) to mucosal (0 mM Cl) across the epithelium. Therefore, in the presence of an apical membrane Cl^−^/HCO_3_^−^ exchanger one will see a rise in pHi as HCO_3_^−^ enters the cell in exchange for Cl^−^. In the absence of a Cl^−^/HCO_3_^−^ exchanger, one would expect to see little or no change in pHi with mucosal Cl^−^ removal because there is no HCO_3_^−^ transport pathway. Our data confirm that IL-17A induces a Cl^−^/HCO_3_^−^ exchange mechanism at the apical membrane of normal HBE cells that is not present in untreated cells as IL-17A-treated cells but not controls respond to removal of mucosal Cl^−^ with a rise in pHi (Figure 3b). As predicted, this change is reversible with replacement of mucosal Cl^−^.

To test the hypothesis that this pathway required HCO_3_^−^, we performed the same experiment in the absence of CO_2_/HCO_3_^−^ with solutions buffered to pH 7.4 using HEPES. In these experiments, there was no rise in pHi with Cl^−^ removal, suggesting that HCO_3_^−^ entry was responsible for the observed rise in pHi. Nonetheless, we observed a small decrease in pHi with Cl^−^ replacement. One possible explanation for this change in pHi is that the IL-17A-induced Cl^−^/HCO_3_^−^ exchange pathway can also exchange Cl^−^ for OH^−^, as has been shown for Pendrin [27]. We note that baseline pH in the CO_2_/HCO_3_^−^-free conditions was higher than that in cells in the presence of CO_2_/HCO_3_^−^, presumably because lack of CO_2_ entry into the cells resulted in less H^+^ generation from carbonic anhydrase. It is also possible, therefore, that we did not see a rise in pHi with Cl^−^ removal from the mucosal solution because the resistance to HCO_3_^−^ entry in the presence of elevated pHi was greater than the driving force for Cl^−^ exit from the cell. A second possibility is that the assay itself failed to detect a rise in pHi because the starting pHi was close to the top of the linear range of detection according to our calibration. However, we note that SNARF dyes demonstrate fluorescence changes at up to pH 9 according to the support material (http://tools.lifetechnologies.com/content/sfs/manuals/mp01270.pdf).In either case above, our data would underestimate Cl^−^/HCO_3_^−^ exchange with Cl^−^ removal and replacement. Because of these limitations, our data taken together are strongly suggestive of Cl^−^/HCO_3_^−^ exchange, but do not establish an absolute dependence on the presence of HCO_3_^−^.

To test the hypothesis that the IL-17A-induced Cl^−^/HCO_3_^−^ exchange pathway was Pendrin, we adapted a previously published method for siRNA inhibition of Pendrin expression [30]. We were successful at reducing, but not completely eliminating Pendrin mRNA and protein expression in anti-Pendrin siRNA-treated cells. This residual expression is likely correlated with the small residual response in pHi to Cl^−^ removal from the mucosal solution seen in the treated cells (Figure 5).

Despite our experimental paradigm, in which we manipulate the direction of Pendrin exchange to transport HCO_3_^−^ into the cell, we hypothesize that under physiological conditions in the airway Pendrin mediates HCO_3_^−^ secretion. This is supported by our previous measurements of surface pH in IL-17A treated HBE cells [20] and by microelectrode impalement studies performed under physiological ionic conditions in which the resting membrane potential of HBE cells is close to the equilibrium potential for Cl^−^ and more negative than that for HCO_3_^−^ [38]. Therefore, under physiological conditions one would anticipate that there would be a greater driving force for HCO_3_^−^ secretion compared with Cl^−^ secretion and Pendrin would facilitate net HCO_3_^−^ secretion across the apical membrane. This conclusion is also supported by the finding that pH_ASL_ is elevated during pathophysiological states where Pendrin has been found to be elevated, including chronic bronchitis and during viral infections [39].

Our data suggest that Pendrin is part of the airway epithelial response to IL-17A. Previously, Pendrin was shown to be part of the airway epithelial response to IL-13 [30]. More recently it has been demonstrated that Th17 and Th2 skewed cytokine profiles are found in stable CF patients preceding the detection of Pseudomonas aeruginosa infection [40]. It remains to be seen whether Th17 and Th2 cytokines have a synergistic effect on Pendrin expression. Our RNA sequencing data suggest little difference in the ability of IL-13 and IL-17 to promote Pendrin RNA expression in HBE cells. While such studies may provide further insight into the role of Pendrin under different inflammatory conditions, drawing conclusions with respect to relative potency may be quite difficult to control because of the multiple variables such as expression and density of receptors signaling machinery that would need to be accounted for, even while the system itself may be changing (see below).

Heterologous expression of Pendrin in fisher rat thyroid (FRT) cells confers cAMP-independent SCN^−^ transport [30]. Such SCN^−^ transport is linked to innate immunity through the activity of lactoperoxidase (LPO) that catalyzes the formation of the antibacterial compound hypothiocyanite (OSCN^−^) from SCN^−^ and hydrogen peroxide (H_2_O_2_) [41]. Similarly, IL-4 increases SCN^−^ transport in HBE cells [30]. Moreover, at least 2 different laboratories have established a direct link between HCO_3_^−^ and antibacterial defenses. In particular, *Sa* grown in the presence of HCO_3_^−^ is significantly more susceptible to antimicrobial peptides than *Sa* grown in medium of the same pH that lacks HCO_3_^−^ [17]. Similarly, in rat prostate, HCO_3_^−^ secretion, but not pH, is critical for bacterial killing [42]. Taken together, these findings suggest that Pendrin may have a role in airways host defense.

Others have suggested that HCO_3_^−^ secretion is critical for the normal secretion and/or unfolding of secreted mucins [43], [44]. This raises the hypothesis that Pendrin expression may be linked to mucin or mucus biology in airway epithelial cells, a hypothesis supported by data demonstrating the role of Pendrin in HCO_3_^−^ secretion from Calu-3 cells, a model of submucosal gland serous cells [45]. Others have shown in mice that virally-mediated over-expression of Pendrin in lung is sufficient for airways mucin production [46]. Our immunofluorescence data raise the possibility that in IL-17A treated cells Pendrin is predominantly, if not exclusively, expressed in non-ciliated cells. Moreover, there appear to be fewer ciliated cells in IL-17A treated monolayers, though we did not undertake statistical analysis to determine if this is definitively the case. Such an effect has been demonstrated previously for IL-13 [47]. Taken together, these data are consistent with a role for Pendrin in the pathophysiology of mucus hyperplasia and metaplasia seen in chronic inflammatory disorders [48]. There is growing evidence that Pendrin plays a role in the host response to infection and inflammation. However, many of the investigations have taken place in cultured epithelial cells. And while differentiated epithelial cells are a good representation of the native epithelium, one must use caution when extending in vitro findings to whole tissue, organ, and animal physiology.

An example for why such caution is warranted is the apparent lack of lung disease in Pendred syndrome patients. This may reflect either relatively low levels of Pendrin in the lung in the absence of inflammation, or the ability of other HCO_3_^−^ transporters, such as CFTR or other SLC26A family members, to compensate for lack of Pendrin in the airways. This may also reflect that absence of Pendrin is protective against some forms of pulmonary disease, a suggestion for which there is evidence in the literature. For example, Pendrin null mice demonstrate attenuated inflammatory responses and reduced airways reactivity in an allergen-induced model of asthma [49]. This response was attributable to increased ASL depth in response to IL-13 stimulation of Pendrin-deficient airway epithelial cells. The precise mechanism of Pendrin regulation of ASL remains unknown and may be directly related to Pendrin, indirectly related to Pendrin through other ion transporters, or to some other effect of Pendrin deficiency not yet identified [49]. Further investigation in physiologically relevant Pendrin null model systems is warranted to determine what role, if any, Pendrin has in airways physiology or pathophysiology and the mechanisms by which it affects cell biology.

## Supporting Information

Figure S1 **Relative potency of interferon-γ (Ifnγ), IL-13, and IL-17A to induce Pendrin in HBE cells.** We note that each condition represents n = 2 donors, so statistical analysis is not appropriate. In these donors, IL-17 and IL-13 appear to induce Pendrin to a greater extent than does Ifn-γ.
(TIF)

Figure S2 **Immunofluorescence detection of Pendrin in IL-17A-treated HBE cells (50 ng/ml, 48 h).** All images: Pendrin is pseudocolored red; Type IV Tubulin marking cilia is pseudocolored green. Top row: Pendrin antibody, clone E-20 (Santa Cruz): **A.** Vehicle controls, **B.** IL-17A-treated cells, **C.** IL-17A-treated cells in the presence of blocking peptide (Santa Cruz, specific for clone). Middle row: Pendrin antibody, clone G-19 (Santa Cruz): **D.** Vehicle controls, **E.** IL-17A-treated cells, **F.** IL-17A-treated cells in the presence of blocking peptide (Santa Cruz, specific for clone). Bottom row: No Pendrin antibody: **G.** Secondary antibodies used alone (note: same secondary antibodies were used for immunofluorescence experiments).
(TIF)

Methods S1 **This section provides details on real-time PCR calculations, microscopy solutions and siRNA transfections.**
(DOCX)
