# Supplementary figures and images for: IL-17A Induces Pendrin Expression and Chloride-Bicarbonate Exchange in Human Bronchial Epithelial Cells
Source: PLoS One. 2014 Aug 20;9(8):e103263. doi: 10.1371/journal.pone.0103263 (PMC4139276; doi:10.1371/journal.pone.0103263)

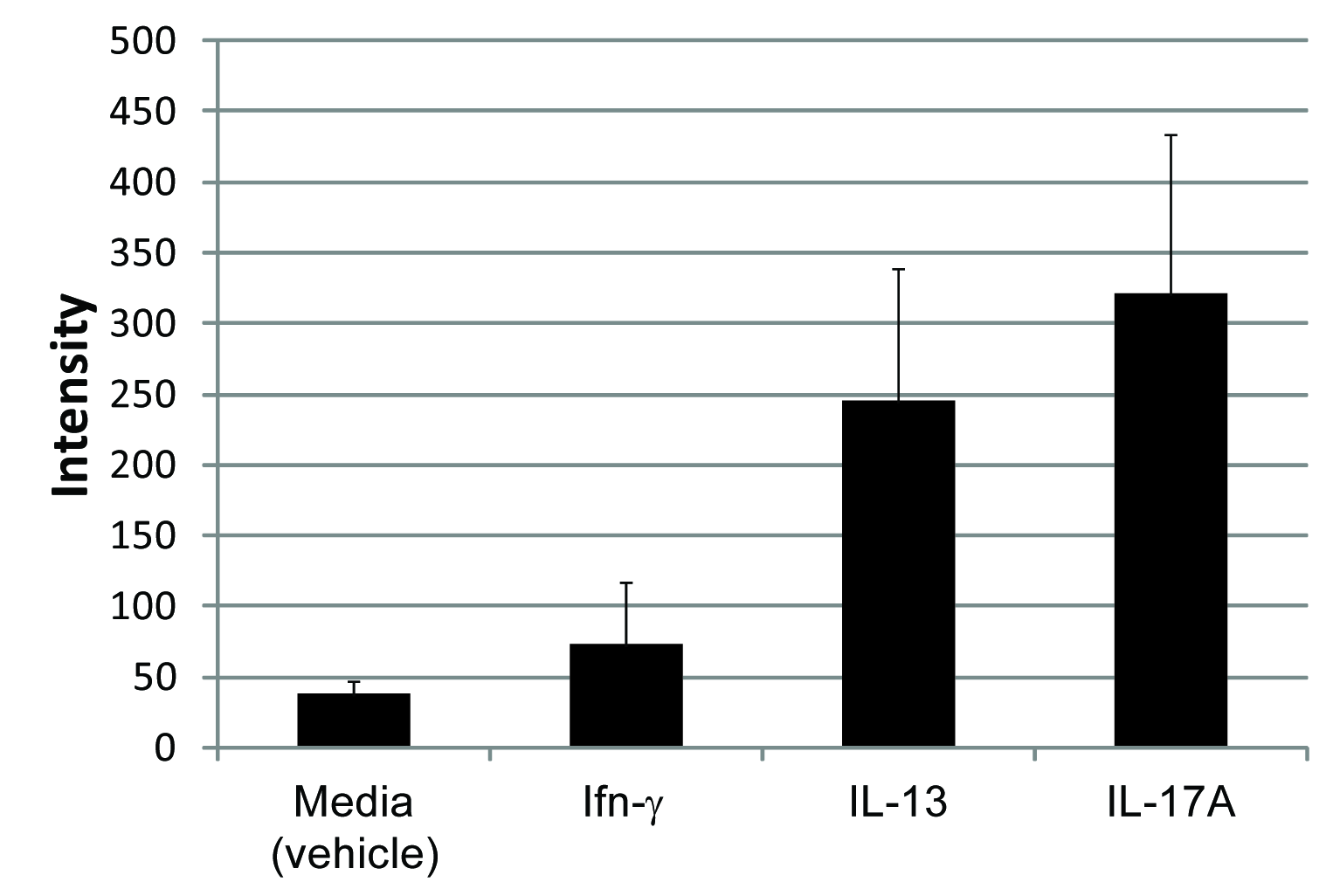

Supplement: Figure S1 — Relative potency of interferon-γ (Ifnγ), IL-13, and IL-17A to induce Pendrin in HBE cells. We note that each condition represents n = 2 donors, so statistical analysis is not appropriate. In these donors, IL-17 and IL-13 appear to induce Pendrin to a greater extent than does Ifn-γ. (TIF) [file pone.0103263.s001.tif]

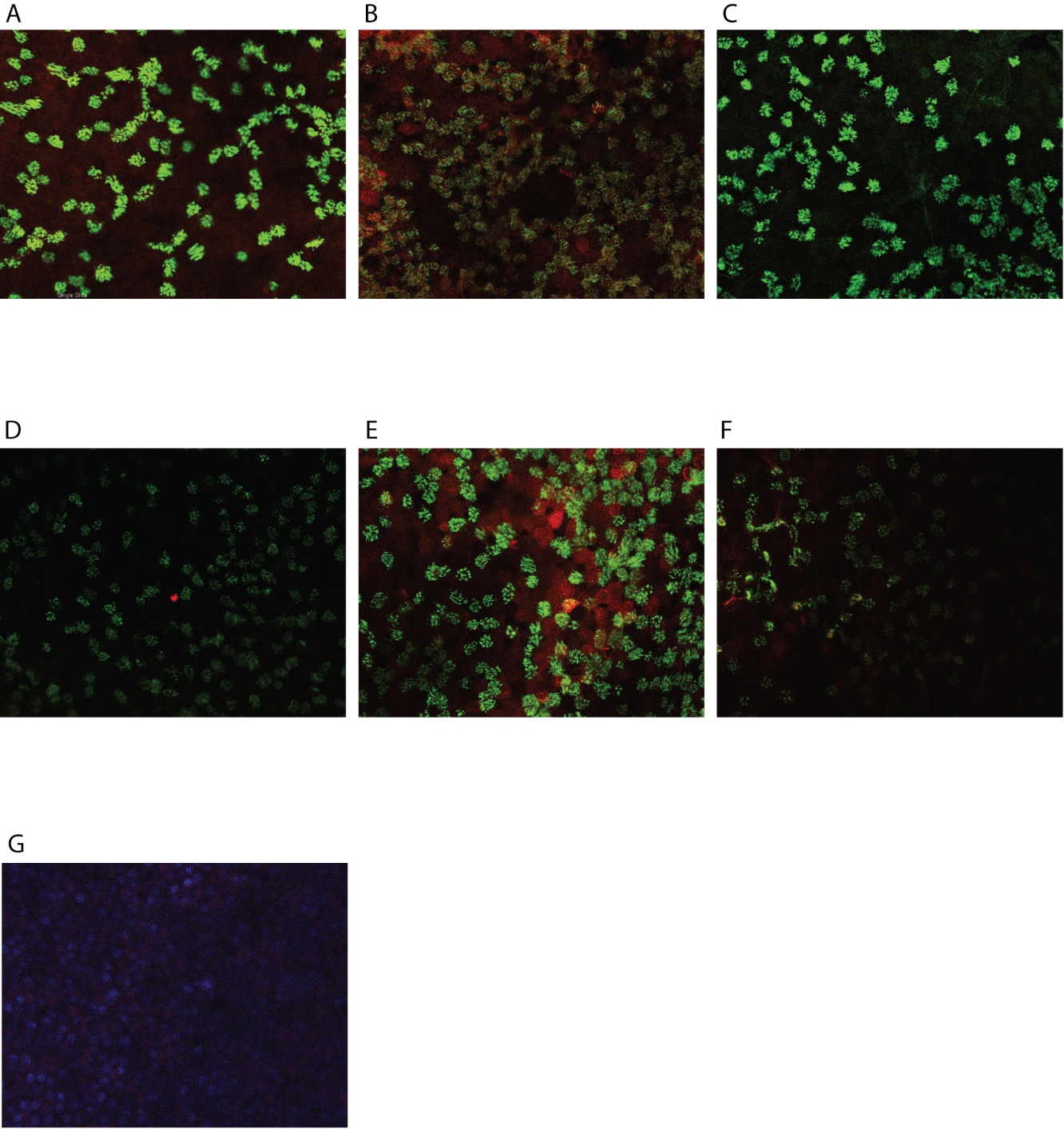

Supplement: Figure S2 — Immunofluorescence detection of Pendrin in IL-17A-treated HBE cells (50 ng/ml, 48 h). All images: Pendrin is pseudocolored red; Type IV Tubulin marking cilia is pseudocolored green. Top row: Pendrin antibody, clone E-20 (Santa Cruz): A. Vehicle controls, B. IL-17A-treated cells, C. IL-17A-treated cells in the presence of blocking peptide (Santa Cruz, specific for clone). Middle row: Pendrin antibody, clone G-19 (Santa Cruz): D. Vehicle controls, E. IL-17A-treated cells, F. IL-17A-treated cells in the presence of blocking peptide (Santa Cruz, specific for clone). Bottom row: No Pendrin antibody: G. Secondary antibodies used alone (note: same secondary antibodies were used for immunofluorescence experiments). (TIF) [file pone.0103263.s002.tif]
