# Supplementary material for: IL-17A Induces Pendrin Expression and Chloride-Bicarbonate Exchange in Human Bronchial Epithelial Cells
Source: PLoS One. 2014 Aug 20;9(8):e103263. doi: 10.1371/journal.pone.0103263 (PMC4139276; doi:10.1371/journal.pone.0103263)
Supplement: Methods S1 — This section provides details on real-time PCR calculations, microscopy solutions and siRNA transfections. (DOCX) [file pone.0103263.s003.docx]

**Methods S1**

**Real-time PCR Calculations:** Real-time PCR was performed using a Step One Real-time PCR machine (Applied Biosystems), and data were output using manufacturer’s software. For each sample, a delta threshold cycle (ΔCt) was calculated by subtracting the threshold cycle for GAPDH from the threshold cycle for Pendrin. These values were then used to calculate 2^-ΔCt^, which is termed the coefficient of variation (CV) by the authors of reference 21. The CV values obtained for the vehicle controls were then averaged to determine the mean CV for comparison. The CV values for each sample were then divided by the mean for the vehicle controls to obtain the relative Pendrin expression in each sample. These values were then averaged and the standard error of the mean calculated for graphing.

**Microscopy solutions:** For CO_2_/HCO_3_^-^-containing experiments, the serosal aspect of the cells rested in solution containing 120 mM NaCl, 25 mM NaHCO_3_, 3.3 mM KH_2_PO_4_, 0.8 mM K_2_HPO_4_, 1.2 mM MgCl_2_, 1.2 mM CaCl_2_, and 10 mM glucose. The mucosal solution was either the same (120 Cl) or one in which all chloride was replaced by equimolar gluconate (0 Cl). Solutions were continuously gassed with 95% O_2_ / 5% CO_2_. For CO_2_/HCO_3_^-^-free experiments, the 120 Cl solution contained: 120 mM NaCl, 25 mM Na Gluconate, 3.3 mM KH_2_PO_4_, 0.8 mM K_2_HPO_4_, 1.2 mM MgCl_2_, 1.2 mM CaCl_2_, 10 mM glucose, and 10 mM HEPES, pH 7.4, and the 0 Cl solution contained: 145 mM Na Gluconate, 3.3 mM KH_2_PO_4_, 0.8 mM K_2_HPO_4_, 1.2 mM Mg Gluconate, 1.2 mM Ca Gluconate, 10 mM glucose, and 10 mM HEPES, pH 7.4. These solutions were continuously gassed with 100% O_2_.

**Transfection with Small-interfering RNA (siRNA):** Normal human bronchial epithelial cells were transfected with siRNA at the time of transfer to coated Transwell inserts (Costar 3470). Cells were seeded at a density of 1X 10^5^ per Transwell insert in media containing 50% LHC 9 in RPIM-1640 without antibiotics together with 40 μl of Opti-MEM medium (Invitrogen Life technologies) containing preformed complexes of siRNA duplex (100nM) and 0.8 μl of Lipofectamine RNAi MAX .The basolateral side contained 500 μl of LHC9 / RPMI1640 without antibiotics. The transfection reagent was from Invitrogen Life technologies and siRNA transfection was performed according to the manufacturer’s instruction. Stealth RNA interference against human Pendrin (code HSS 107794, HS 107796) were obtained from Invitrogen Life technologies. The negative control, also from Invitrogen, was a non-targeting control siRNA (code 12935-200). After transfection siRNA complexes were removed after 24 h and cells were fed with differentiation media. After 14 days the transected cells were used for experiments.
